# Soil Nutrient Depletion Is Associated with the Presence of Burkholderia pseudomallei

**DOI:** 10.1128/AEM.02538-16

**Published:** 2016-11-21

**Authors:** Viriya Hantrakun, Patpong Rongkard, Malinee Oyuchua, Premjit Amornchai, Cherry Lim, Vanaporn Wuthiekanun, Nicholas P. J. Day, Sharon J. Peacock, Direk Limmathurotsakul

**Affiliations:** a Mahidol-Oxford Tropical Medicine Research Unit, Faculty of Tropical Medicine, Mahidol University, Bangkok, Thailand; b Centre for Tropical Medicine and Global Health, Nuffield Department of Clinical Medicine, Churchill Hospital, University of Oxford, Oxford, United Kingdom; c Department of Medicine, University of Cambridge, Cambridge, United Kingdom; d London School of Hygiene and Tropical Medicine, London, United Kingdom; e Department of Tropical Hygiene, Faculty of Tropical Medicine, Mahidol University, Bangkok, Thailand; Rutgers, The State University of New Jersey

## Abstract

Burkholderia pseudomallei is a soil-dwelling bacterium and the cause of melioidosis, which kills an estimated 89,000 people per year worldwide. Agricultural workers are at high risk of infection due to repeated exposure to the bacterium. Little is known about the soil physicochemical properties associated with the presence or absence of the organism. Here, we evaluated the soil physicochemical properties and presence of B. pseudomallei in 6,100 soil samples collected from 61 rice fields in Thailand. The presence of B. pseudomallei was negatively associated with the proportion of clay, proportion of moisture, level of salinity, percentage of organic matter, presence of cadmium, and nutrient levels (phosphorus, potassium, calcium, magnesium, and iron). The presence of B. pseudomallei was not associated with the level of soil acidity (*P* = 0.54). In a multivariable logistic regression model, the presence of B. pseudomallei was negatively associated with the percentage of organic matter (odds ratio [OR], 0.06; 95% confidence interval [CI], 0.01 to 0.47; *P* = 0.007), level of salinity (OR, 0.06; 95% CI, 0.01 to 0.74; *P* = 0.03), and percentage of soil moisture (OR, 0.81; 95% CI, 0.66 to 1.00; *P* = 0.05). Our study suggests that B. pseudomallei thrives in rice fields that are nutrient depleted. Some agricultural practices result in a decline in soil nutrients, which may impact the presence and amount of B. pseudomallei bacteria in affected areas.

**IMPORTANCE** Burkholderia pseudomallei is an environmental Gram-negative bacillus and the cause of melioidosis. Humans acquire the disease following skin inoculation, inhalation, or ingestion of the bacterium in the environment. The presence of B. pseudomallei in soil defines geographic regions where humans and livestock are at risk of melioidosis, yet little is known about the soil properties associated with the presence of the organism. We evaluated the soil properties and presence of B. pseudomallei in 61 rice fields in East, Central, and Northeast Thailand. We demonstrated that the organism was more commonly found in soils with lower levels of organic matter and nutrients, including phosphorus, potassium, calcium, magnesium, and iron. We also demonstrated that crop residue burning after harvest, which can reduce soil nutrients, was not uncommon. Some agricultural practices result in a decline in soil nutrients, which may impact the presence and amount of B. pseudomallei bacteria in affected areas.

## INTRODUCTION

Melioidosis, an infectious disease caused by the Gram-negative bacterium Burkholderia pseudomallei, is an important global public health threat. An estimated 165,000 cases of human melioidosis occur each year worldwide, of which 89,000 cases (54%) die (1). The disease is highly endemic in Southeast Asia and northern Australia (2) and is predicted to be endemic but grossly underreported in many tropical and subtropical countries (1, 3). The crude case fatality rate for melioidosis ranges from 14% to 40% and may be as high as 70% in cases given suboptimal antibiotic therapy (4–6). No licensed vaccine for melioidosis is currently available.

B. pseudomallei is a free-living organism found in soil and water (2), and humans acquire the disease following skin inoculation, inhalation, or ingestion of the bacterium in the environment (7). In tropical developing countries, most patients are agricultural workers (typically rice farmers) with frequent contact with soil and water. Evidence-based guidelines for the prevention of melioidosis recommend that residents and visitors to areas endemic for melioidosis avoid direct contact with soil and water and wear protective gear, such as boots and gloves, when in direct contact with soil and environmental water (7, 8). However, rubber boots are hot and make walking difficult in muddy rice fields, and rubber gloves are also hot and difficult to use while planting rice (9). As a result, many rice farmers continue to work in rice fields without protective gear and are at high risk for melioidosis.

The presence of B. pseudomallei in soil defines geographic regions where humans and livestock are at risk of melioidosis, but knowledge of the environmental factors associated with the presence of the organism in the natural setting is poor and conflicting. Laboratory studies using sterile soil showed that B. pseudomallei grows well in soil with a high percentage of moisture (10–12), high level of iron (13), optimal acidity (pH 4 to 8) (11, 13), and high salinity (up to 4.2 deciSiemens [dS]/m) (13). In contrast, two cross-sectional studies in the natural environment in northern Australia and Northeast Thailand found that the presence of B. pseudomallei was negatively associated with the level of iron in soil (14, 15), and a recent modeling study and an experimental field study suggested that the presence of B. pseudomallei was not associated with soil acidity (1, 12). Furthermore, both negative and positive correlations between the presence of B. pseudomallei and soil salinity have been reported (1, 12, 15). Land use can affect the biodiversity of organisms in soil (16), but there is currently no information on the association between the presence of B. pseudomallei and agricultural practices.

Here, we report the findings of a large cross-sectional environmental survey to determine the physicochemical characteristics of soil associated with the presence of B. pseudomallei in three regions in Thailand where melioidosis is considered to be highly endemic (Northeast and East) or nonendemic (Central). Our findings extend the understanding of soil properties related to environmental B. pseudomallei.

## MATERIALS AND METHODS

### Study area.

East, Central, and Northeast Thailand consist of 7, 21, and 20 provinces that cover 34,381, 93,005, and 168,854 km^2^, and have estimated populations in 2013 of 3.9, 18.7, and 23.3 million, respectively (17). Northeast Thailand is a plateau surrounded by mountain ranges, and most of the arable land consists of tropical sandy soil. East Thailand is characterized by short mountain ranges alternating with alluvial plains. Central Thailand is a large plain consisting of clay soil. Rice farming is the predominant form of agriculture in all three regions. In Thailand, for administrative purposes, each province is subdivided into districts, subdistricts, communes, and villages. The majority of the population in all three regions lives in rural settings, and most adults are engaged in agriculture, particularly rice farming. In 2013, the percentages of land used for agriculture were 57%, 48%, and 60% in East, Central, and Northeast Thailand, respectively (18).

**FIG 1:**
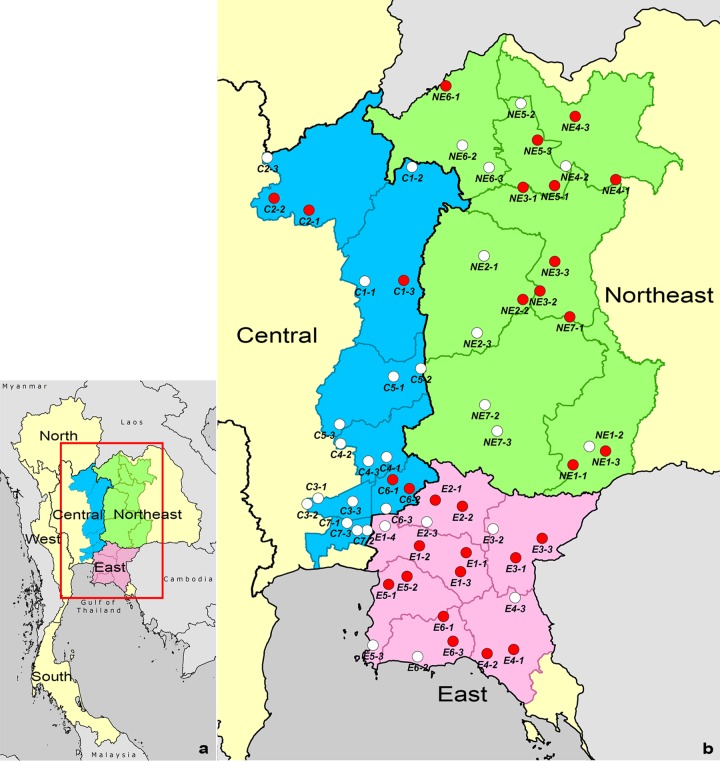
Distribution of B. pseudomallei in Central, East, and Northeast Thailand. (a) Map of Thailand. (b) Location of the 61 rice fields evaluated. Red and white circles, culture positive and culture negative for B. pseudomallei, respectively. Province codes represent Phetchabun (C1), Phitsanulok (C2), Pathum Thani (C3), Saraburi (C4), Lopburi (C5), Nakhon Nayok (C6) and Bangkok (C7) in Central Thailand, Chachoengsao (E1), Prachinburi (E2), Sa Kaeo (E3), Chanthaburi (E4), Chonburi (E5) and Rayong (E6) in the East, and Burirum (NE1), Chaiyaphum (NE2), Khon Kaen (NE3), Udon Thani (NE4), Nong Bua Lam Phu (NE5), Loei (NE6), and Nakhon Ratchasima (NE7) in the Northeast. Maps drawn using ArcGIS 10.2 (ESRI, Redlands, CA, USA).

To evaluate environmental factors associated with the presence of B. pseudomallei, we selected six, seven, and seven adjacent provinces in East, Central, and Northeast Thailand, respectively (Fig. 1). Three villages per province were randomly selected. Randomization was performed using Stata version 14.0 (StataCorp LP, College Station, TX). Soil sampling was performed in one rice field per one village. Rice fields were selected as sampling sites, since rice farming is a major risk factor for melioidosis (9). The sampled fields were those that had been used for rice farming for at least 12 months prior to the sampling date. Written informed permission was obtained from landowners prior to sampling.

The study protocol was approved by the ethics committee of the Faculty of Tropical Medicine, Mahidol University (MUTM 2013-021-01) and the Oxford Tropical Research Ethics Committee, University of Oxford (OXTREC 1013-13).

### Soil sampling.

Soil sampling in East, Central, and Northeast Thailand was performed during the dry season (from April to June) in 2013, 2014, and 2015, respectively. We used the consensus guidelines for environmental sampling described by the Detection of Environmental Burkholderia pseudomallei Working Party (DEBWorP) (19). In brief, each rice field was divided into a grid system, in which 100 sampling points (10 by 10) were plotted 2.5 m apart. At each sampling point, around 30 g of soil was removed from the base of a 30-cm hole, placed in a ziplock bag, and kept at ambient temperature and protected from sunlight. We recorded the location of sampled fields using the EpiCollect application (www.epicollect.net; Imperial College, London, United Kingdom) (20). All soil samples were processed within 48 h of collection for the identification of B. pseudomallei and for soil physicochemical properties.

### Identification of B. pseudomallei.

Ten grams of soil from each sampling point was mixed with 10 ml of enrichment broth consisting of threonine-basal salt solution plus colistin (TBSS-C50 broth) and incubated at 40°C in air for 48 h. Ten microliters of surface liquid was then subcultured onto Ashdown agar, incubated at 40°C in air, and examined every 24 h for 4 days for bacterial colonies suggestive of B. pseudomallei, which were initially identified on the basis of colony morphotype. This included the characteristic colony morphology (purple, flat, dry, and wrinkled) together with six additional colony morphotypes, as described previously (21). Presumptive colonies were picked from each sample and tested immediately using a specific latex agglutination test for B. pseudomallei-specific capsular polysaccharide (CPS), as previously described (22). For positive colonies, susceptibility to amoxicillin-clavulanic acid and arabinose assimilation were determined as previously described (23). B. pseudomallei was defined based on the combination of colony morphology, positive latex agglutination test, susceptibility to amoxicillin-clavulanic acid, and negative arabinose assimilation (23).

### Soil properties.

One kilogram of soil from each sampling field was made by aggregating 100 soil samples (10 g per each sampling point) and evaluating for four main properties, as follows: (i) physical properties of texture (proportion of sand, silt, and clay) and moisture (% [wt/wt]); (ii) acidity and salinity, as determined by pH, lime requirement (to adjust soil acidity; kg/100 m^2^), and electrical conductivity (dS/m); (iii) chemical properties of total nitrogen (milligrams per kilogram), available phosphorus (milligrams per kilogram), exchangeable potassium (milligrams per kilogram), exchangeable calcium (milligrams per kilogram), available magnesium (milligrams per kilogram), extractable sulfur (milligrams per kilogram), total iron (grams per kilogram), total cadmium (milligrams per kilogram), exchangeable sodium (milligrams per kilogram), and cation exchange capacity (centimoles per milligram); (iv) biological related factors of organic matter (% [wt/wt]) and carbon-to-nitrogen ratio (C:N ratio) (see Table S5 in the supplemental material). All soil properties were evaluated by iLab Asia (Kanchanaburi, Thailand) except for total iron and total cadmium, which were evaluated by Central Laboratory (Bangkok, Thailand). Both laboratories were registered with the Ministry of Agriculture Thailand as standardized national soil testing laboratories.

### Agricultural practices.

A closed-end interviewee-based questionnaire was used to collect the information about agricultural practices. For illiterate participants, the questionnaire was read to the participant and completed by trained research staff in accordance with their responses. Questions included fertilizer used and rice field management (before planting and after harvest) in the 12 months before the sampling date.

### Sample size calculation.

To determine the optimal sample size, we performed a pilot study of soil sampling in four rice fields in Chachoengsao province, East Thailand. Three of four rice fields (75%) were culture positive for B. pseudomallei. We calculated that 60 rice fields (3 rice fields per province) were needed to determine environmental factors associated with B. pseudomallei with a power of 80% at an alpha error of 5%.

### Statistical analysis.

The outcomes of interest were positivity of B. pseudomallei in rice fields and its association with soil properties. Binary and continuous variables were compared by using Fisher's exact test and a Mann-Whitney test, respectively. Soil properties associated with the presence of B. pseudomallei were evaluated using univariable and multivariable logistic regression. The final multivariable logistic regression models were developed using a purposeful selection method (24). Sensitivity analysis was conducted using region-stratified analysis. We also used ordered logistic regression to evaluate the association between soil properties and the quantity of B. pseudomallei. The number of positive sampling points for B. pseudomallei within a rice field was used to represent the distribution of B. pseudomallei quantity in the field. The Spearman correlation coefficient was used to evaluate the correlation between soil properties. All statistical tests were performed using Stata version 14.0 (StataCorp LP, College Station, TX). The final database with the data dictionary is publicly available online (http://dx.doi.org/10.6084/m9.figshare.3298180).

## RESULTS

### Distribution of B. pseudomallei in Northeast, East, and Central Thailand.

Of 6,100 soil samples collected from 61 rice fields (100 soil samples per rice field), 1,046 samples were culture positive for B. pseudomallei (Fig. 1). A total of 30 of 61 rice fields (49%) had at least one sampling point that was culture positive for the organism. The percentages of rice fields that were culture positive for B. pseudomallei were 57% (12 of 21 rice fields), 68% (13 of 19 rice fields), and 24% (5 of 21 rice fields) in Northeast, East, and Central Thailand, respectively. The percentages of rice fields that were culture positive for B. pseudomallei in the Northeast and East was higher than that in Central Thailand (57% versus 24%, respectively, *P* = 0.06; and 68% versus 24%, respectively, *P* = 0.01), while the percentages were not significantly different between the Northeast and East (57% versus 68%, *P* = 0.53).

For the rice fields that were culture positive for B. pseudomallei, the median numbers of positive sampling points were 53 (range, 2 to 98), 16 (range, 1 to 81), and 1 (range, 1 to 63) in Northeast, East, and Central Thailand, respectively (see Table S1 in the supplemental material). The median numbers of positive sampling points in the Northeast and East were both higher than that in Central Thailand (*P* = 0.01 and 0.002), while the numbers for the East and Northeast were not significantly different (*P* = 0.61).

### Characteristics of soil and agricultural practices.

An overall comparison of soil properties among three regions showed that soil from Central Thailand had the highest median percentage of clay (53%), followed by the Northeast (45%) and East (32%). Soil acidity (pH) varied considerably, ranging from very acidic (pH 4.9) to carbonate-rich soil (pH 8.1), but these values were not significantly different between the three regions (*P* = 0.68). Soil salinity, as determined by electrical conductivity and expressed in deciSiemens per meter, was very low in all fields sampled (<2.0 dS/m).

Farmers were interviewed about land management before and after rice planting (including the fertilizer used and crop residue burning before and after harvest) in the 12 months before the sampling date. Of 61 rice fields evaluated, 54 (89%) fields were treated with chemical fertilizer, 17 (28%) fields were treated with organic fertilizer made from plant material, 22 (36%) fields were treated with organic fertilizer made from animal dung, and 39 (64%) fields were treated with biological fertilizer, such as effective microorganisms. Owners of 24 (39%) rice fields burned their fields between rice planting seasons. The median percentage of organic matter in fields with a history of burning was not significantly lower than that of others (0.81 versus 0.84% [wt/wt], respectively; *P* = 0.82).

### Association between soil physicochemical properties and B. pseudomallei.

**FIG 2:**
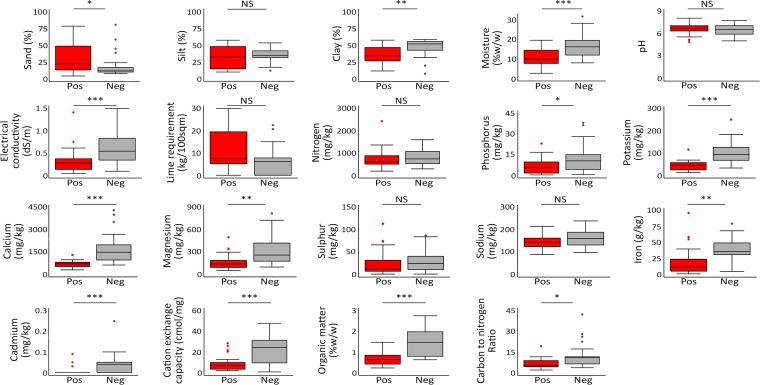
Soil physicochemical properties associated with the presence of B. pseudomallei. Box-and-whisker plots indicate median, interquartile range, and distribution of the data. Dots indicate the outliers (data located outside 1.5 times of interquartile range) (41). Red and gray boxes represent rice fields that are culture positive (Pos) and culture negative (Neg) for B. pseudomallei, respectively. *, *P* ≤ 0.05; **, *P* ≤ 0.01; ***, *P* ≤ 0.001. NS, not significant.

We found that the presence of B. pseudomallei was associated with soil nutrient depletion (Fig. 2; see also Table S2 in the supplemental material). Presence of the organism was negatively associated with the percentage of soil moisture (*P* < 0.001), level of soil salinity (*P* = 0.001), presence of cadmium (*P* < 0.001), and levels of multiple nutrients, including available phosphorus (*P* = 0.03), exchangeable potassium (*P* < 0.001), exchangeable calcium (*P* = 0.001), available magnesium (*P* = 0.002), and total iron (*P* = 0.002). Levels of overall nutrients and total nutrient fixing capacity of soil determined by organic matter and cation exchange capacity, respectively, were also negatively associated with the presence of B. pseudomallei (both *P* < 0.001). The carbon-to-nitrogen ratio, which is used to determine how easily bacteria can decompose organic material in soil, was also negatively associated with the presence of B. pseudomallei (*P* = 0.01). Presence of the organism was positively associated with the proportion of sand (*P* = 0.02), negatively associated with the proportion of clay (*P* = 0.002), and was not associated with the proportion of silt (*P* = 0.68). The presence of B. pseudomallei was not associated with soil acidity (*P* = 0.54) or agricultural practices. Many soil physicochemical properties were strongly correlated (see Table S3 in the supplemental material).

We used multivariable logistic regression analysis and found that the presence of B. pseudomallei was negatively associated with the percentage of organic matter (OR, 0.06; 95% CI, 0.01 to 0.47; *P* = 0.007), level of salinity (OR, 0.06; 95% CI, 0.01 to 0.74; *P* = 0.03), and level of soil moisture (OR, 0.81; 95% CI, 0.66 to 1.00; *P* = 0.05) (Table 1). A sensitivity analysis was conducted by including region as a stratification variable, which gave comparable results.

**TABLE 1:** Soil physicochemical properties associated with the presence of B. pseudomallei in a multivariable logistic regression model

Soil physicochemical characteristic	Adjusted odds ratio (95% CI)^*a*^	*P* value
Organic matter (% [wt/wt])	0.06 (0.01–0.47)	0.007
Electrical conductivity (dS/m)	0.06 (0.01–0.74)	0.03
Moisture (%)	0.81 (0.66–1.00)	0.05

a CI, confidence interval.

In addition, we also used ordered logistic regression to further evaluate the association between the distribution of B. pseudomallei quantity in rice fields and soil physicochemical factors. We observed that the number of sampling points culture positive for B. pseudomallei was also negatively associated with the percentage of organic matter (OR, 0.06; 95% CI, 0.01 to 0.32; *P* = 0.001), level of soil moisture (OR, 0.78; 95% CI, 0.66 to 0.91; *P* = 0.002), and level of salinity (OR, 0.07; 95% CI, 0.01 to 0.53; *P* = 0.01) (see Table S4 in the supplemental material).

## DISCUSSION

The results of our large environmental study demonstrated an association between the presence of B. pseudomallei and soil nutrient depletion in rice fields in Thailand. Negative associations between the presence of B. pseudomallei and nutrient levels in the soil were observed for each of the nutrients evaluated (with the exception of total nitrogen, exchangeable sodium, and extractable sulfur) and for organic matter and cation exchange capacity, which represent levels of overall nutrients and total nutrient fixing capacity of soil, respectively. This is also supported by the negative association between the presence of B. pseudomallei and the level of salinity, which could also represent the level of soil nutrients in the environment (12). Our findings are important because nutrients in the soil are affected by agricultural practices, and crop residue burning after harvest is not uncommon in Thailand and many other tropical countries. There is strong evidence to show that burning can reduce soil nutrients by eliminating crop residues and soil organisms present on the soil surface (25). Poor agricultural practices could impact the presence and amount of B. pseudomallei. This suggests that changes in agricultural practice and improvement of soil nutrient content might also be essential to reduce the distribution of B. pseudomallei and incidence of melioidosis.

Our study also highlights the difference between findings from experimental soil inoculated with B. pseudomallei, environmental studies in small areas where melioidosis is endemic, and this large environmental study. For example, soil moisture was positively associated with the presence of B. pseudomallei in experimental soil studies (10–12) and environmental studies of small areas where melioidosis is endemic (26, 27). It has been postulated that B. pseudomallei can move from deeper soil layers to the surface during the rainy season and rising water table, where it may then multiply (28). Our study shows that soil in Northeast Thailand (where B. pseudomallei is abundant in soil) is mostly sandy soil with a low level of organic matter and moisture, while soil in Central Thailand (where B. pseudomallei is less abundant) is mostly clay soil with high levels of organic matter and moisture. This is also supported by a recent finding of the presence of B. pseudomallei in a desert region outside the wet tropics in northern Australia (29).

Organic matter in soil contains vital nutrients and influences the diversity and biological activity of soil organisms (25). The negative association between soil organic matter and the presence of B. pseudomallei is consistent with two previous environmental studies in northern Australia (15) and Northeast Thailand (30), which showed that the level of organic carbon was negatively associated with the presence of B. pseudomallei. The level of organic carbon is a measure of the carbon contained within the soil organic matter. It is possible that soils with high organic matter have high biotic stress, because abundant soil microorganisms are competing for substrates, water, or growth factors (31). This competition may inhibit the survival or growth of B. pseudomallei. This is supported by an environmental study showing that low microbial density in soil is associated with the presence of B. pseudomallei (27, 32), and that Bacillus amyloliquefaciens extracted from soil samples can inhibit the growth of B. pseudomallei (32). It is also possible that depletion of individual nutrients, such as iron, supports the growth of B. pseudomallei, which has a range of mechanisms to persist in low-iron environments (33). An additional possibility is that environmental stress selects for persister cells of B. pseudomallei, as has been recently shown for Pseudomonas aeruginosa under nutrient-limited conditions and in biofilms (34). B. pseudomallei is taken up by amoebae, which *in vitro* are associated with survival in the presence of disinfecting agents and antimicrobial drugs (35, 36) and may represent an additional survival advantage for B. pseudomallei in soil nutrient depletion.

Our findings suggest that extremely low levels of salinity (such as <0.1 dS/m) may be an indirect measure of nutrient depletion in rice fields. This is because soil salinity, as estimated by measuring electrical conductivity, represents soluble salts of soil nutrients, including sodium, chloride, magnesium, calcium, potassium, and nitrate. Our finding is consistent with an experimental study in northern Australia (12), which showed that B. pseudomallei grew well in soil with low electrical conductivity (0.1 dS/m) but could not survive in commercial soil, which has a high level of organic based compost and high electrical conductivity (0.7 dS/m). Although a recent modeling study proposed a positive association between salinity level and presence of B. pseudomallei, this estimation was based on soil salinity for all land (undisturbed land, agricultural land, sports fields, etc.) with an electrical conductivity ranging from 0 to >20 dS/m (1). It is also possible that the effect of salinity in rice fields may be different from that in nonrice fields. For example, rice fields may be intentionally flooded and drained repeatedly to reduce salinity to a very low level (<2.0 dS/m) (37), and this could lead to the loss of water-soluble nutrients from the soil (38–40).

B. pseudomallei can survive well in soil under laboratory conditions with pH ranging from 4 to 8 (13), and our study supports the lack of association between the presence of B. pseudomallei and pH.

A limitation of our study is that soil sampling was only performed during the dry season over a period of 3 years. We chose to sample during the dry season to control for variation in the presence of B. pseudomallei and soil physicochemical properties associated with seasonal changes. Recent environmental studies showed that soil properties were not different between the dry and wet seasons (14), and that changes in the presence of B. pseudomallei in the soil with very low salinity level (<2.0 dS/m) measured over 3 years were minimal (12). It is possible that the presence of B. pseudomallei in rice fields would have been generally higher if the study was conducted during the rainy season. Although the difference in the percentage of organic matter between fields with and without a history of burning was not observed in our study, this could be because of the cross-sectional study design or other confounding factors. For example, some fields were burned more than 12 months before the study was conducted.

In summary, our large cross-sectional environmental survey has shown that the presence of the important human pathogen B. pseudomallei is associated with nutrient-depleted rice fields. Further investigations are required to evaluate whether changes in agricultural practices could effectively enhance soil nutrients, and whether these could reduce the distribution of B. pseudomallei in rice fields.

## Supplementary Material

Supplemental material
