# Supplementary material for: Soil Nutrient Depletion Is Associated with the Presence of Burkholderia pseudomallei
Source: Appl Environ Microbiol. 2016 Nov 21;82(24):7086–92. doi: 10.1128/AEM.02538-16 (PMC5118919; doi:10.1128/AEM.02538-16)
Supplement: Supplemental material [file supp_82_24_7086__index.html]

Supplemental material 

# Soil Nutrient Depletion Is Associated with the Presence of Burkholderia pseudomallei

## Supplemental material

**Files in this Data Supplement:**

- Supplemental file 1 -

  Number of culture-positive samples for *B. pseudomallei* (Table S1), association between soil properties and *B. pseudomallei* (Table S2), correlation among soil properties (Table S3), association between soil properties and quantity of *B. pseudomallei* distribution (Table S4), and methods used to determine soil properties (Table S5).

  PDF, 351K
