# Supplementary material for: Soil Nutrient Depletion Is Associated with the Presence of Burkholderia pseudomallei
Source: Appl Environ Microbiol. 2016 Nov 21;82(24):7086–92. doi: 10.1128/AEM.02538-16 (PMC5118919; doi:10.1128/AEM.02538-16)
Supplement: Supplemental material [file AEM.02538-16_zam999117556so1.pdf]

Supplemental material

Supplement to: Soil nutrient depletion is associated with the presence of *Burkholderia pseudomallei*

Viriya Hantrakun[1], Patpong Rongkard[1], Malinee Oyuchua[1], Premjit Amornchai[1], Cherry Lim[1], Vanaporn Wuthiekanun[1], Nicholas PJ Day[1,2], Sharon J. Peacock[1,3,4], Direk Limmathurotsakul[1,2,5*]

[1] Mahidol-Oxford Tropical Medicine Research Unit, Faculty of Tropical Medicine, Mahidol University, Thailand

[2] Centre for Tropical Medicine and Global Health, Nuffield Department of Clinical Medicine, Churchill Hospital, University of Oxford, United Kingdom

[3] Department of Medicine, Cambridge University, United Kingdom

[4] London School of Hygiene and Tropical Medicine, London, United Kingdom

[5] Department of Tropical Hygiene, Faculty of Tropical Medicine, Mahidol University, Thailand

TABLE S1 Number of culture positive samples for *B. pseudomallei* in 61 rice fields

Regions	Province	Field *	Number of sampling points culture positive for *B. pseudomallei* (per 100 sampling points)
Northeast	Burirum	NE1-1	87
		NE1-2	0
		NE1-3	97
	Chaiyaphum	NE2-1	0
		NE2-2	98
		NE2-3	0
	Khon Kaen	NE3-1	72
		NE3-2	94
		NE3-3	35
	Udon Thani	NE4-1	40
		NE4-2	0
		NE4-3	28
	Nong Bua Lam Phu	NE5-1	58
		NE5-2	0
		NE5-3	48
	Loei	NE6-1	4
		NE6-2	0
		NE6-3	0
	Nakhon Ratchasima	NE7-1	2
		NE7-2	0
		NE7-3	0
East	Chachoengsao	E1-1	39
		E1-2	8
		E1-3	43
		E1-4	0
	Prachinburi	E2-1	10
		E2-2	6
		E2-3	0
	Sa Kaeo	E3-1	17
		E3-2	0
		E3-3	81
	Chanthaburi	E4-1	16
		E4-2	1
		E4-3	0
	Chonburi	E5-1	3
		E5-2	32

Regions	Province	Field *	Number of sampling points culture positive for ***B. pseudomallei*** (per 100 sampling points)
		E5-3	0
	Rayong	E6-1	1
		E6-2	0
		E6-3	57
Central	Phetchabun	C1-1	0
		C1-2	0
		C1-3	3
	Phitsanulok	C2-1	1
		C2-2	1
		C2-3	0
	Pathum Thani	C3-1	0
		C3-2	0
		C3-3	0
	Saraburi	C4-1	0
		C4-2	0
		C4-3	0
	Lopburi	C5-1	0
		C5-2	0
		C5-3	0
	Nakhon Nayok	C6-1	63
		C6-2	1
		C6-3	0
	Bangkok	C7-1	0
		C7-2	0
		C7-3	0

* Each rice field was divided into a grid system, in which 100 sampling points (10 by 10) were plotted 2.5 m apart. At each sampling point, 10 g of soil at 30 cm depth was collected and cultured for *B. pseudomallei*.

TABLE S2 Soil physicochemical properties associated with the presence of *B. pseudomallei*

Soil physicochemical characteristics	Rice fields positive for *B. pseudomallei*[1] (n=30)	Rice fields negative for *B. pseudomallei*[1] (n=31)	Crude odds ratio (95% CI)	p value
**Physical factors**				
• Sand (%)	22.4 (4.3-78.8)	12.0 (7.8-81.0)	1.04 (1.01-1.07)	0.02
• Silt (%)	32.2 (9.7-57.2)	34.5 (11.7-53.2)	0.99 (0.95-1.03)	0.68
• Clay (%)	34.1 (11.5-57.6)	51.9 (7.3-58.6)	0.93 (0.88-0.97)	0.002
• Moisture (%w/w)	9.9 (2.5-19.5)	16.1 (7.9-31.8)	0.78 (0.67-0.90)	<0.001
**Acidity and salinity factors**				
• pH	6.8 (4.9-8.1)	6.6 (5.0-7.8)	1.23 (0.63-2.40)	0.54
• Electrical conductivity (dS/m)	0.3 (0.04-1.4)	0.5 (0.1-1.5)	0.22 (0.09-0.53)	0.001
• Lime requirement (kg/100sqm)	7.5 (0-30)	6.2 (0-22.5)	1.00 (1.00-1.01)	0.10
**Chemical factors**				
• Total nitrogen (mg/kg)	598 (175-2,442)	731 (278-1,601)	0.99 (0.98-1.01) [2]	0.41
• Available phosphorous (mg/kg)	5 (0.2-23)	10 (0.4-38)	0.35 (0.15-0.83) [2]	0.02
• Exchangeable potassium (mg/kg)	45 (11-115)	93 (32-252)	0.52 (0.37-0.74) [2]	<0.001
• Exchangeable calcium (mg/kg)	673 (272-1,289)	1474 (602-4,326)	0.93 (0.90-0.97) [2]	0.001
• Available magnesium (mg/kg)	134 (44-498)	255 (93-823)	0.92 (0.87-0.97) [2]	0.003
• Extractable sulphur (mg/kg)	12 (0-114)	24 (0-88)	0.94 (0.77-1.15) [2]	0.55
• Exchangeable sodium (mg/kg)	144 (89-217)	161 (98-241)	0.87 (0.75-1.01)[2]	0.07
• Total iron (Fe; g/kg)	12 (0.6-96)	36 (4- 79)	0.95 (0.92-0.98)	0.002
• Total cadmium				
○ not detected	25 fields (83%)	11 fields (35%)	1.0	
○ detected	5 fields (17%)	20 fields (65%)	0.11 (0.03-0.37)[3]	<0.001
• Cation exchange capacity (cmol/mg)	6.7 (2.0-28.5)	24.4 (0.6-47.9)	0.89 (0.84-0.95)	<0.001
**Biological related factors**				
• Organic matter (%w/w)	0.6 (0.2-1.5)	1.5 (0.6-2.8)	0.02 (0.003-0.17)	<0.001
• Carbon to nitrogen ratio	5.4 (2.0-19.3)	11.0 (3.6-42.6)	0.81 (0.70-0.94)	0.01

[1] Median (range) unless other are specified.

[2] Odds ratio for any increase of 100 mg/kg in nutrient.

TABLE S3 Correlation coefficients among soil physicochemical properties in the East, Central and Northeast Thailand

	Sand	Silt	Clay	pH	LR[1]	OM[1]	EC[1]	TotalN[1]	AvailP[1]	ExchK[1]
Sand	1									
Silt	-0.74***	1								
Clay	-0.77***	0.13	1							
pH	0.12	-0.03	-0.15	1						
LR[1]	0.22	-0.29*	-0.05	-0.49***	1					
OM[1]	-0.25*	-0.01	0.38**	-0.10	-0.18	1				
EC[1]	-0.14	-0.09	0.29*	-0.10	-0.11	0.35**	1			
TotalN[1]	-0.13	-0.08	0.27*	-0.15	0.14	0.35**	0.18	1		
AvailP[1]	-0.17	0.16	0.11	-0.04	-0.01	0.37**	0.11	0.28*	1	
ExchK[1]	-0.27*	-0.11	0.51***	-0.26*	-0.16	0.63***	0.44***	0.31*	0.40**	1
ExchCa[1]	-0.19	-0.07	0.35**	-0.06	-0.36**	0.57***	0.29*	0.10	0.17	0.64***
AvailMg[1]	-0.27*	0.02	0.38**	-0.14	-0.28*	0.47***	0.19	0.16	0.003	0.47***
ExtrS[1]	-0.20	0.17	0.14	-0.20	-0.01	0.14	0.21	0.14	0.16	0.17
ExchNa[1]	-0.38**	0.26*	0.32*	-0.23	0.03	0.26*	0.12	0.16	0.24	0.29*
CEC[1]	-0.31*	-0.06	0.51***	-0.25	-0.12	0.76***	0.34**	0.27*	0.18	0.69***
Moisture	-0.46***	0.16	0.53***	-0.27*	-0.18	0.55***	0.23	0.26*	0.20	0.60***
CNRatio[1]	0.003	0.001	-0.01	0.05	-0.24	0.57***	0.04	-0.36**	0.28*	0.27*
CD[1]	-0.25*	0.12	0.26*	0.07	-0.13	0.63***	0.15	0.09	0.24	0.32*
Iron[1]	-0.38**	0.08	0.48***	-0.16	0.004	0.63***	0.11	0.21	0.27*	0.42***

Statistically significant *$p \leq 0.05$, **$p \leq 0.01$ and ****$p \leq 0.001$

	ExchCa[1]	AvailMg[1]	ExtrS[1]	ExchNa[1]	CEC[1]	Moisture	CNRatio[1]	CD[1]	Iron[1]
ExchCa[1]	1								
AvailMg[1]	0.69***	1							
ExtrS[1]	-0.01	0.19	1						
ExchNa[1]	0.26*	0.37**	0.21	1					
CEC[1]	0.62***	0.68***	0.24	0.33**	1				
Moisture	0.39**	0.31*	0.10	0.33**	0.55***	1			
CNRatio[1]	0.38**	0.27*	0.05	0.09	0.39**	0.24	1		
CD[1]	0.41***	0.54***	0.04	0.36**	0.54***	0.22	0.46***	1	
Iron[1]	0.29*	0.29*	0.09	0.29*	0.46***	0.53***	0.34**	0.48***	1

Statistically significant *p≤0.05 , **p≤0.01 and ****p≤0.001

[1]Abbreviations used for soil properties: lime requirement (LR), electrical conductivity (EC), total Nitrogen (TotalN), available phosphorous (AvailP), exchangeable potassium (ExchK), exchangeable calcium (ExchCa), available magnesium (AvailMg), extractable sulphur (ExtrS), total iron (Iron), total cadmium (CD), exchangeable sodium (ExchNa) and cation exchange capacity (CEC), organic matter (OM) and carbon to nitrogen ratio (CNRatio)

TABLE S4 Association between soil physicochemical properties and quantity of *B. pseudomallei* distribution in rice field determined by ordered logistic regression

Soil physicochemical characteristics	Adjusted odds ratio (95% CI)	p value
Organic matter (%w/w)	0.06 (0.01-0.32)	0.001
Moisture (%)	0.78 (0.66-0.91)	0.002
Electrical conductivity (dS/m)	0.07 (0.01-0.53)	0.01

TABLE S5 Soil physicochemical properties methods used and normal range

Soil physicochemical characteristics	Method & Definition	Normal range
**Physical factors**		
• Percentage of sand, silt and clay	Hydrometer method classified by particles size: sand (0.05– 2 mm), silt (0.002 – 0.05 mm) and clay (<0.002 mm)	Combination of sand, silt and clay is 100%
• Moisture (%w/w)	Gravimetric method	Not available
**Acidity and salinity factors**		
• pH	pH meter method: Acidity and alkalinity of the soil	Extremely acid <4.5,<br>Very acid soil 4.5-5.5,<br>Mild acid 5.6-6.5,<br>Neutral 6.6–7.3,<br>Mild alkaline 7.4-7.8,<br>Alkaline 7.9-8.4,<br>Very alkaline 8.5-9.0, Extremely alkaline 9.0[2]
• Electrical conductivity (dS/m)	EC meter method: soluble salt content in the soil	Very low (non-saline) 0-2,<br>Mild salinity 2-4,<br>saline soil 4-8,<br>high 8-16,<br>very high ≥16[2]
• Lime requirement (kg/100sqm)	Woodruff buffer method	Not available
**Chemical factors**		
• Total nitrogen (mg/kg)	Kjeldahl method	Not available
• Available phosphorous (mg/kg)	Bray II method	Very low <6,<br>low 6-12,<br>average 13-25,<br>high 26-50,<br>very high >50 mg/kg[2]
• Exchangeable potassium (mg/kg)	Automated Flame photometric method	Very low <16,<br>low 16-30,<br>average 31-60,<br>high 60-120,<br>very high >120 mg/kg[2]
• Exchangeable calcium (mg/kg)	Flame photometic method	Not available
• Available magnesium (mg/kg)	Flame photometic method	Not available
• Extractable sulphur (mg/kg)	Turbidimetric method	Not available
• Exchangeable sodium (mg/kg)	Flame photometic method 6020	Not available
• Total iron (Fe; g/kg)[3]	Based on US EPA method 6101B	Not available
• Total cadmium (mg/kg) [3]	Based on US EPA method 6020	<37 mg/kg[1]

Soil physicochemical characteristics	Method & Definition	Normal range
• Cation exchange capacity (cmol/kg)	Filtration method: The number of exchangeable cations per dry weight that a soil is capable of holding, at a given pH value, and available for exchange with the soil water solution.	>15 cmol/kg[2]
**Biological related factors**		
• Organic matter (%w/w)	Walkley and black method.	very low <0.5%,<br>low 0.5-1.5%,<br>average 1.5-2.5%,<br>somewhat high 2.5-3.5%,<br>high 3.5-4.5%,<br>very high >4.5%[2]
• Carbon to nitrogen ratio	Walkley and black method/Kjeldahl method: A ratio of the mass of carbon to the mass of nitrogen in a substance.	Not available

[1] Pollution Control Department (PCD), Ministry of Science Technology and Environment, Thailand

[2] Land Development Department (LDD), Ministry of Agriculture and Cooperatives, Thailand

[3] Iron and cadmium were tested by Central Laboratory (Bangkok, Thailand).
